# Attrition Rates in Multiple Myeloma - A Report from indian Patients at A Tertiary Cancer Center

**DOI:** 10.1007/s12288-025-02188-4

**Published:** 2025-12-05

**Authors:** Dhyey Mishra, Aditya Nair, Jash Shah, Saswata Saha, Lingraj Nayak, Alok Shetty, Prashant Tembhare, Ajmat Khan, Nishant Jindal, Sumeet Mirgh, Anant Gokarn, Sachin Punatar, Sweta Rajpal, Gaurav Chatterjee, Nikhil Patkar, Papagudi Subramanian, Dhanalaxmi Shetty, Hemani Jain, Vasundhara Patil, Sayak Choudhary, Ameya Puranik, Hasmukh Jain, Manju Sengar, Navin Khattry, Bhausaheb Bagal

**Affiliations:** 1 Seth GS Medical College and KEM Hospital, Mumbai, Parel India; 2 Department of Medical Oncology, Tata Memorial Centre, Homi Bhabha National Institute, Mumbai, India; 3 Department of Hemato-pathology, Advanced Centre for Training, Research and Education in Cancer (ACTREC), Tata Memorial Centre, Homi Bhabha National Institute, Mumbai, NaviMumbai India; 4 Department of Cancer Cytogenetics, Advanced Centre for Training, Research and Education in Cancer (ACTREC), Tata Memorial Centre, Homi Bhabha National Institute, Mumbai, NaviMumbai India; 5 Department of Radiodiagnosis,Tata Memorial Centre, Homi Bhabha National Institute, Mumbai, India; 6 Department of Nuclear Medicine, Tata Memorial Centre, Homi Bhabha National Institute, Mumbai, India

**Keywords:** Multiple myeloma, Newly diagnosed multiple myeloma, Attrition rates, Real world data, Indian cohort, Overall survival, Treatment outcomes

## Abstract

Because of its relapsing-remitting nature, patients with multiple myeloma [1,2] require extended therapy with chemotherapy, novel agents, autologous stem cell transplants (ASCT), monoclonal antibodies and/or immune therapies for optimal outcomes. Attrition during treatment can negatively impact long-term outcomes and factors influencing the same may be important clues to achieve optimal patient outcomes. This study aims to examine attrition rates and the factors influencing them.We analysed 244 consecutive newly diagnosed patients of multiple myeloma from January 2017 to December 2017 registered at Tata Memorial Hospital, Mumbai. The patient factors such as age, comorbidities, clinical and laboratory evaluations were recorded along with the lines of therapy (LOTs) received, best response and use of ASCT. Attrition was defined as failure to receive a subsequent line of therapy despite progression of the disease or death from disease according to similar stringent definitions used in similar studies elsewhere. Factors influencing attrition were analyzed using the chi-square test for categorical variables, and either the t-test or logistic regression for continuous variables. An odds ratio greater than 1 and a p-value less than 0.05 were considered as statistically significant. The median age of study cohort was 57.11 years, with 66.4% being male. CRAB (Hypercalcemia, renal dysfunction, anemia, bony lesion) features were noted in most patients with following frequencies: 42 (17.3%) patients with hypercalcemia, 70 (28.9%)-renal dysfunction, 15 (6.2%) of the patients received dialysis,139 (56.9%) patients had anaemia at presentation and 213 (87.2%) patients had bony lesions. R-ISS was available for 199 patients with 19 (9.5%), 128 (64.3%) and 45 (22.6%) of them having R-ISS I, II, and III respectively. 26.2% patients had high-risk cytogenetics (as per the IMWG consensus criteria 2016) at presentation. The most common 1st line therapy given was bortezomib, cyclophosphamide and dexamethasone combination (77.5% of patients). 8.2% of patients had stem cell transplants done & 22.63% patients had received maintenance post first-line therapy. The median progression free survival (PFS) and overall survival (OS) was 3.337 years and 5.043 (Range = 4.143–5.943) years respectively.The median OS in the attrited group was 2.87 years (Range 2.37–3.37 years) while those with attrition to second line therapy showed even poorer median OS of 1.08 years (Range 0.67–1.49 years). The attrition rates for individual lines of therapy were 28.6% for the second LOT, 34.2% for the 3rd LOT, and 45.3% for the subsequent LOTs. The overall attrition rate at the end of follow up was 62.9% (141 patients). Among the factors analysed for attrition, on univariate analysis higher age, ECOG scores of 2 or more, renal dysfunction at presentation, ISS stage 3, and not receiving ASCT showed a significantly higher likelihood of attrition. On multivariate analysis (Overall regression model significant with *p* < 0.001) only having an ECOG of 2 or higher was significantly associated with higher risk of attrition. Our study shows a high proportion of patients do not receive subsequent lines of therapy in our clinical setting highlighting the importance of first-line treatment including ASCT and maintenance. Most effective therapies should be used in earlier lines so that patients can benefit from all available treatment modalities.

## Introduction

Multiple myeloma (MM) is a clonal proliferation of plasma cells characterized by abnormal secretion of monoclonal paraproteins, resulting in end organ damage [1, 2]. Initial treatment includes induction, consolidation with ASCT for eligible patients, and maintenance therapy [3]. However, the disease remains an incurable, chronic relapsing disease, often requiring additional therapeutic interventions. Treatment of relapsed diseases gets increasingly complicated due to advancing age, increasing cumulative end organ damage, emergence of refractory clones leading to decreasing benefits with subsequent lines of therapy [4, 5].

Adherence to therapy declines as the number of therapy lines increases [6], impacting patient outcomes. Different countries have reported different attrition rates [7–9] and multiple factors such as age, comorbidities, disease status, autologous stem cell transplant (ASCT) eligibility, patient reported QoL(quality of life) have shown to affect attrition in their particular population groups. Despite having a significant prevalence of multiple myeloma, similar studies are lacking in the Indian setting [10]. This study aims to fill the gap in attrition data and thus help plan the treatment course better, identify key factors affecting attrition and possibly overcome it to prevent patient dropouts.

## Methodology

### Study Design and Data Sources

The study is a retrospective observational analysis of patients above 18 years of age who are newly diagnosed with multiple myeloma between January 2017 to December 2017 at Tata Memorial Hospital, Mumbai. Approval was obtained from the institutional ethics committee (IEC) at Tata Memorial Hospital (TMH) (IEC approval no: 901026). Data was collected from the electronic medical records (EMR) of TMH, Mumbai, India. An attempt to telephonically contact patients lacking adequate follow up data was made and information regarding current status and further treatment was inquired in patients who responded. Patients diagnosed with solitary plasmacytoma, plasmablastic lymphoma, smoldering myeloma, MGUS, amyloidosis, plasma cell leukemia, POEMS syndrome and relapsed cases were excluded from the study.

### Variables Recorded

The variables recorded from the EMR data included demographic information, clinical characteristics, laboratory parameters, diagnostic workup, and treatment characteristics. For each line of therapy, remission status, best response and the attrition rate were recorded. Outcomes of patients for each line of therapy was recorded as per the IMWG response assessment criteria [11]. Patients who had no subsequent visits and did not respond to call follow up for more than 6 months were considered as Lost to follow up (LTFU).

Attrition was defined as failure to receive a subsequent line of therapy despite progression of the disease or death from disease according to similar stringent definitions used in similar studies elsewhere. [9, 12–14].

### Statistical Analysis

Statistical analysis was performed using SPSS 23.0 software (SPSS Inc., Chicago, IL).

Demographic and baseline characteristics were reported using descriptive statistics where percentages and counts were used for categorical variables while means, medians, standard deviations, and range was used for continuous variables.

Attrition for each subsequent line of therapy was reported as percentages. Factors influencing attrition were analyzed using the chi-square test for categorical variables, and either the t-test or logistic regression for continuous variables. An odds ratio greater than 1 and a p-value less than 0.05 were considered as statistically significant.

Overall survival (OS) and progression free survival (PFS) were calculated using Kaplan Meier estimate. Significant factors affecting OS and PFS were determined using univariate analysis with a log rank test. Cox regression was used for multivariate analysis of the factors affecting OS and PFS for significance.

## Results

Data of 244 patients were analysed for demographic data, treatment characteristics, overall survival, and progression free survival, excluding patients diagnosed with solitary plasmacytoma, plasmablastic lymphoma, smoldering myeloma, MGUS, amyloidosis, plasma cell leukemia, POEMS syndrome, and relapsed cases (*N* = 40). For attrition analysis 224 patients were analysed, excluding patients who have not received a single line of therapy (*N* = 20).

**Figure:**
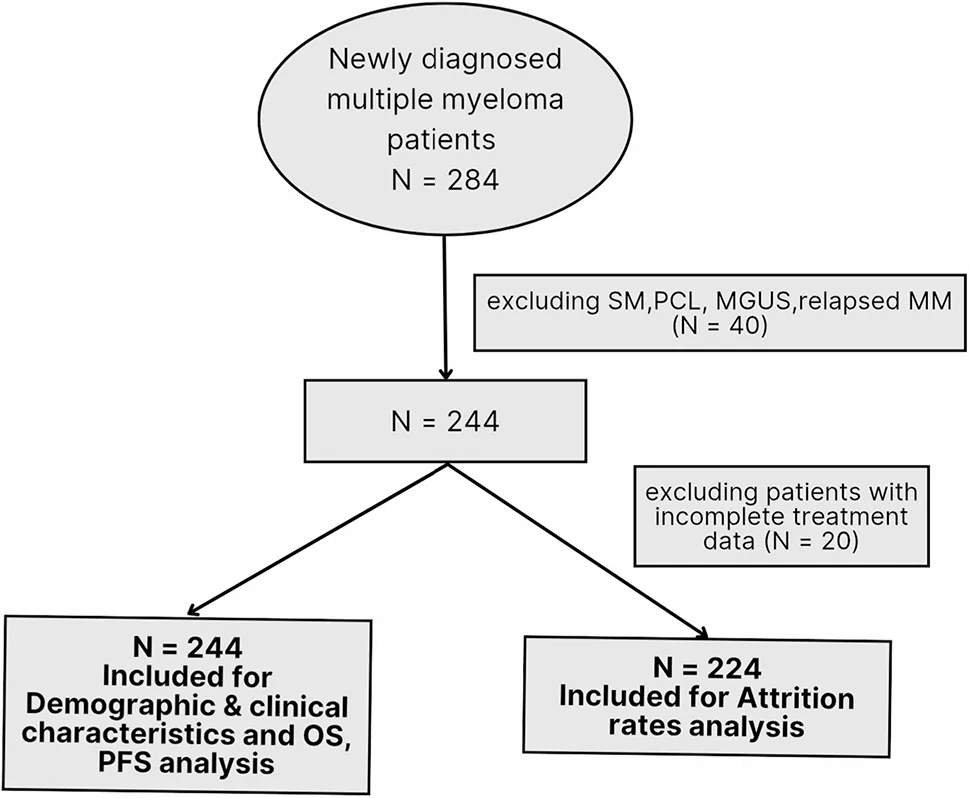

### Presentation Characteristics

The median age of the study population at diagnosis was 57.11 years (range 26 to 79 years) and 162 (66.4%) were male. Cytogenetics revealed hyperdiploidy in 98 out of 234 tested for the same (38.5%) and high-risk IgH translocation {t(4;14), (14;16), (14;20)} was reported in 42 (17.9%) patients while 1q gain was present in 30 (12.7%) patients. 17p/TP53 del was detected in 7 (2.8%) patients.High risk cytogenetics (as per the IMWG consensus criteria 2016) [15] was present in 58 (24.7%). R-ISS was available for 199 patients with 19 (9.5%), 128 (64.3%) and 45 (22.6%) of them having R-ISS I, II, and III respectively. The presentation characteristics of the study are summarised in Table 1.

**Table 1:** Demographic and disease characteristics of patients at diagnosis

Demographic characteristics (N = 244)	Median (range) or Absolute number	Percentages
Median Age at diagnosis(years)	57.11 (26–79)	
Sex ratio (Male: Female)	162:82	66.4%:33.6%
*ECOG PS*		
<2	123	50.4%
≥2	121	49.59%
*CRAB features*		
Hypercalcemia	42	17.3%
Renal Dysfunction	70	28.9%
Anemia	139	56.9%
Bony Lesions	213	87.2%
*R-ISS (N = 199)*		
I	19	9.5%
II	128	64.3%
III	45	22.6%
Blood parameters	Median (Range)	
Hb	9.5gm/dl (2.8 to 15.8)	
Calcium	9.5mg/dl (5.5 to 16.18)	
LDH	184.5 (58 to 1159)	
Creatinine	1.2mg/dl (0.6 to 21)	
*Cytogenetics (N = 234)*		
Hyperdiploidy	98	38.5%
high risk IgH translocation (t(4;14), (14;16), (14;20))	42	17.9%
1q gain	30	12.7%
17p/TP53 del	7	2.8%

### Treatment Patterns

Triplet regimen remains the treatment of choice as the first line of therapy for NDMM patients of which VCd was used in 189 out of 244 (77.5%) patients, 29 (11.9%) received VRd, 5 (2%) patients received VTd while 17 (7%) patients who were not fit for triplet therapy received doublet therapy (Lenalidomide + Dexamethasone, Bortezomib + Dexamethasone, Melphalan + Prednisolone), and 4% patients received other therapies. The first line of treatment has been summarized in Table 2. The median number of cycles patients received was 9 cycles (range = 1 to 26 cycles). Twenty (8.2%) patients received ASCT. Fifty-five (22.63%) of the patients received maintenance therapy and the median duration of maintenance was 17 months. 158 (64.75%) patients continued to the second line of treatment due to clinical and molecular progression.

The most common second line of therapy were VRd (40.5%) and Lenalidomide + dexamethasone (21.48%). Other second-line therapies included VCd, Pomalidomide + Dexamethasone, MPT, KPd, KRd, Rd, thalidomide + Dexamethasone, and DPD.

**Table 2:** Treatment patterns for newly diagnosed multiple myeloma patients

*First line of treatment*	
VCd	189 (77.5%)
VRd	29 (11.9%)
Others	26 (10.65%)
*Maintenance*	
Received	26 (10.65%)
Not Received	136 (55.96%)
Data not available	53 (21.72%)
*ACST*	
Yes	20 (8.2%)
No	191 (78.6%)
Data not available	33 (13.52%)

### Treatment Outcomes

The overall response rate (ORR) following first-line therapy was highest with VRd at 76.9%, followed by VCd at 67.3%, and Len + Dexa at 63.7%. These findings reflect the superior efficacy of triplet regimens, particularly those incorporating immunomodulatory agents like lenalidomide, over doublet therapies, aligning with outcomes reported in global studies.

### Attrition Analysis

**Fig. 1:**
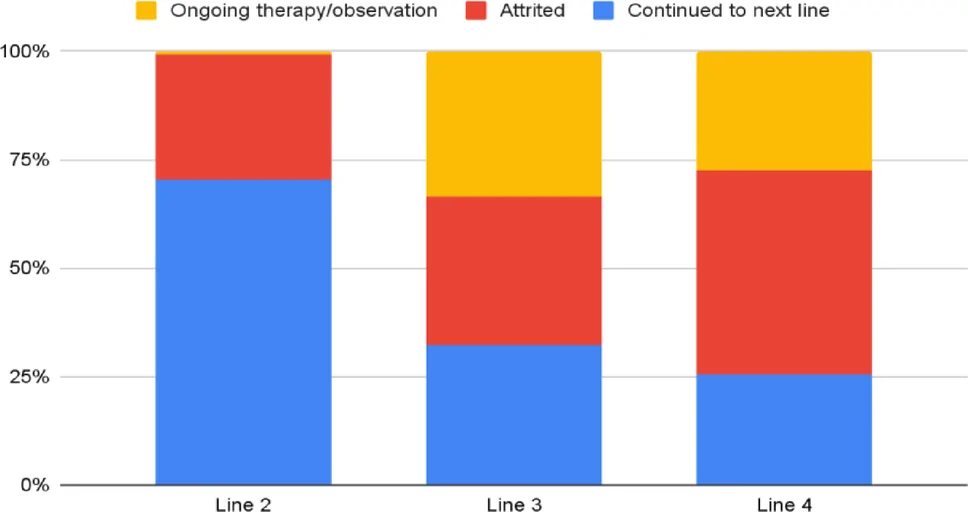
Stacked bar chart showing attrition rates for various LOT

For attrition analysis only patients who received at least one line of therapy were included and a few patients were excluded because their attrition data was inappropriate (*total 224 patients were included*).The attrition rates for individual lines of therapy were 28.6% for the second LOT, 34.2% for the 3rd LOT, and 45.3% for the subsequent LOTs. The overall attrition rate at the end of follow up was 62.9% (141 patients) Fig 1.

After the first line of therapy 70.5%(158 out of 224) patients went on to receive the next line of therapy while the rest had attrited and 0.9% did not require further therapy. Among the patients who received second line of therapy, 32.2% continued to the next line of treatment, 33.5% were under treatment or were under observation and 34.2% had attrited. Among the patients who received the third line of therapy 25.5% patients continued to receive subsequent lines of therapy, 27.4% patients were on the current line of therapy/under observation, and 47.1% patients had attrited. Characteristics of Attrited patients are summarised in Table 3

**Table 3:** Baseline and treatment characteristics of attrited patients

	Attrited (*N* = 141)	Non-Attrited (83)
Age (median, range)	58 (28–79)	53 (27–73)
ECOG 2 or more	84 (59.6%)	32 (38.6%)
Renal dysfunction	50 (35.5%)	15 (18.1%)
ISS stage 3	74 (52.5%)	30 (36.1%)
RISS Stage 3	32 (22.7%)	10 (12%)
High risk cytogenetics	36 (25.5%)	23 (27.7%)
Doublet therapy	9 (6.4%)	3 (3.6%)
ASCT vs. others	6 (4.3%)	14 (16.9%)

Among the factors analysed for attrition, on univariate analysis higher age, ECOG scores of 2 or more, renal dysfunction at presentation, ISS stage 3, and not receiving ASCT showed a significantly higher likelihood of attrition (p- values 0.005, 0.003, 0.011, 0.015, and 0.003 respectively). On multivariate analysis (Overall regression model significant with *p* < 0.001) only having an ECOG of 2 or higher was significantly associated with higher risk of attrition. Summary of factors and their p values are given in Table 4.

Analysing factors affecting attrition to second line therapy showed that apart from ECOG scores, having 17p deletions, and receiving doublet therapy as induction was associated with a higher risk of attrition (p values of 0.042 and 0.014 respectively). On multivariate analysis [Overall regression model significant with *p* = 0.01], doublet therapy showed significant association with attrition to second line treatment (*p* = 0.034).

**Table 4:** Analysis of factors affecting attrition

Parameters	Frequencies/median	Univariate Analysis *p*-value	Multivariate analysis *p*-value
Age	57.11 (26–79)	0.005	0.119
ECOG 2 or more	116/224	0.003	0.005
Renal dysfunction	65/224	0.011	0.49
ISS 3 vs. others	104/213	0.015	0.253
High risk cytogenetics	58/224	0.644	-
Del 17p	7/224	0.38	-
Doublet therapy	11/224	-	-
ASCT vs. others	20/224	0.003	0.108

### Overall Survival

The survival data was collected till May 2023. The median overall survival (OS) for the whole cohort is 5.043 (95% CI = 4.143 to 5.943) years. The median OS for the attrited group is 2.87 years (95% CI = 2.37–3.37 years).

Patients who had attrition to 2nd line of therapy had a poorer median OS, it being 1.08 years (95% CI = 0.67–1.49 years).

**Fig. 2:**
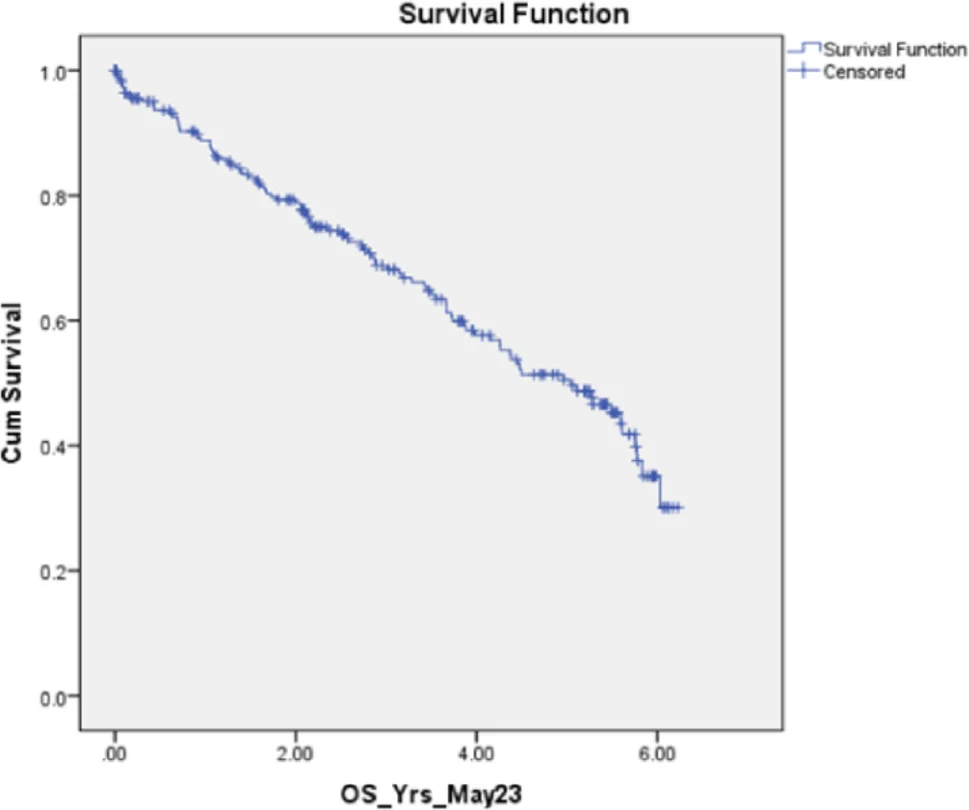
Overall Survival (OS) using Kaplan Meier estimate

**Fig. 3:**
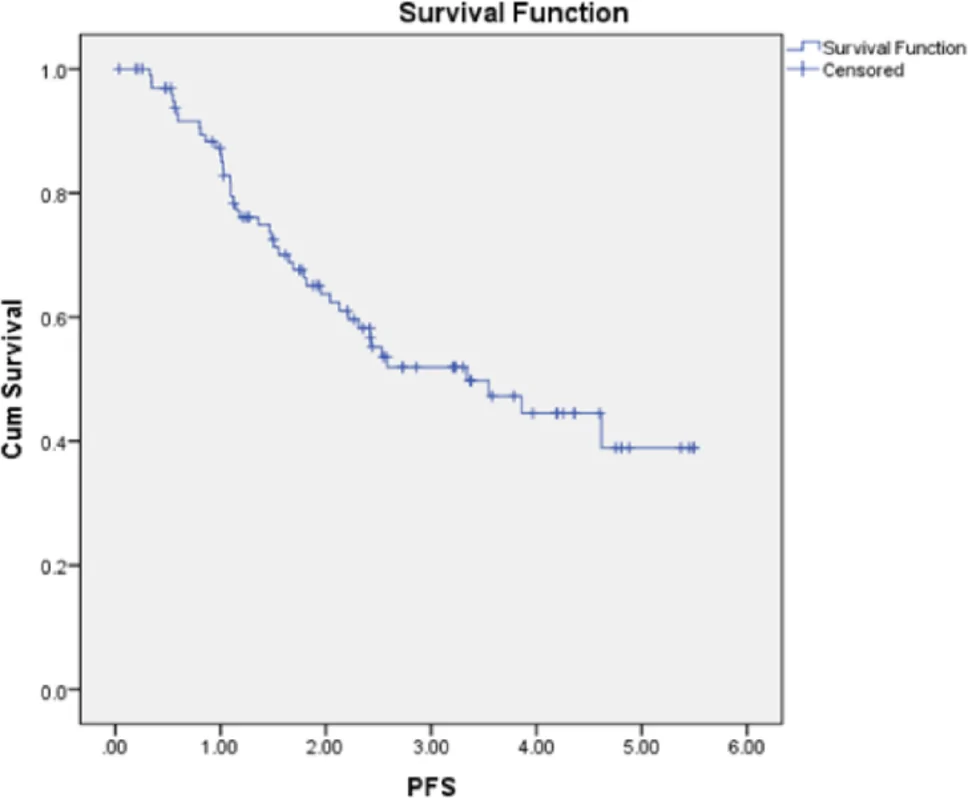
Progression Free Survival (PFS) using Kaplan Meier estimate

The progression free survival (PFS) is 3.337 years for the cohort. Kaplan meier estimate curves for Overall Survival (OS) and Progression Free Survival have been given in Fig. 2 and 3 respectively.

## Discussion

This is a single institutional analysis of patients with Newly Diagnosed Multiple Myeloma (NDMM) diagnosed during a period between January 2017 to December 2017 and treated over the subsequent years till May 2023. This period was selected so as to allow documentation of adequate follow up and outcome duration.

Our study highlights some important demographic and clinical features in Indian patients. The Median age at diagnosis in our cohort was 57.11 years - notably lower than global datasets from Latin America, Spain, United States, Taiwan [8, 12, 13, 16]. This possibly represents the Indian population structure and referral bias towards treatment at tertiary centers for younger multiple myeloma patients. Despite younger age, a high proportion of our patients had comorbidities which related with the globally available data suggesting a younger age of onset of comorbidities [8, 17–21]. The ECOG PS at presentation was ≥ 2 in higher proportion of patients as compared to global data [8, 22]. Among the CRAB features, a higher proportion of patients had bony lesions and renal dysfunction at presentation than reported from western literature.This along with the higher ISS stages in our cohort points towards an aggressive disease course, delayed presentation, and significant organ damage at diagnosis contributing to poor outcomes.

The attrition analysis conducted in this study revealed a concerning finding of high attrition rates with increase in attrition rates with each subsequent line of therapy, rising from 28.6% after the first line to 45.3% after the third line. Despite having a younger patient cohort, these rates are much higher than those reported in studies using similar stringent definitions of attrition [7, 9, 14]. This suggests an aggressive disease phenotype in the population further emphasised by the association of higher ISS stages (*p* < 0.05) and renal impairment (*p* < 0.05) at presentation and the association of poorer performance status with attrition in the cohort.

A range of socioeconomic factors also contributed to the higher attrition rates in our cohort as a substantial group of study population belonged to lower socioeconomic strata registered in general/trust supported categories. Furthermore, many patients reported reasons like financial constraints, lack of accessibility to come to Mumbai due to considerable distance from their primary residence, lack of awareness and illiteracy were also the other factors influencing treatment discontinuation.

Furthermore, treatment modality emerged as a crucial factor influencing attrition, with higher rates of attrition to second line therapy observed in patients receiving doublet (Vd, Rd, Len + dexa) therapy compared to triplet (VCd, VRd, MPT, etc.) therapy, aligning with the known efficacy of triplet regimens in disease management [7, 23–25]. Among the treatment patterns, despite the advantages of VRd regimen, VCd was used as first line therapy in the majority of the patients due to limited access to lenalidomide at the time of study. Patients undergoing ASCT were associated with significantly lower attrition rates in univariate analysis indicating the efficacy of response along with better disease characteristics and follow up rates.

The median OS for our population was 5.043 years and the median PFS was 3.337 years which is lower compared to the data reported in the west [1] As expected, median OS for the attrition group was much lower compared to those continuing treatment and even poorer for patients not receiving the second line of therapy [2.87 years (Range 2.37–3.37 years) and 1.08 years (Range 0.67–1.49 years) respectively.

Higher attrition rates with subsequent lines indicate that most patients do not go on to receive those therapies. This coupled with the findings that delayed presentation, aggressive disease characteristics, and suboptimal initial therapy are the major factors associated with attrition and ultimately overall survival, it is of prime importance that an aggressive therapeutic approach upfront to achieve deeper responses and sustained disease control, which is also documented among various other study cohorts [26, 27].

There are several limitations to be considered in the retrospective analysis. The real world data is a sum of individual treatment decisions made by physicians and differs from controlled and standardized methods used in clinical trials. As this data is collected from a single centre, it becomes difficult to distinguish true attrition from loss of follow up, changing treatment institutes, etc. compared to data availed using large disease specific registries in other countries. The lower number of patients receiving ASCT also hampers the analysis and associations made in this group due to financial reasons [28]. Also the attrition analysis represents patients who have received at least one line of therapy and not the entire NDMM population and must be interpreted in the same context.

These limitations also underscores the need for prospective, multicenter registry data in India to distinguish true attrition from loss to follow-up and better characterise the MM treatment landscape. A more structured referral system and improved access to triplet regimens and ASCT can also reduce attrition and improve outcomes.

A comparison of various studies has been summarized in Table 5.

**Table 5:** Comparison of studies

Study group	Setting	Factor influencing attrition rates
Canadian study	Retrospective study of 5548 patients from CMRG-DB database between Jan 1, 2010 - Dec 31, 2020	Older age
		Shorter time to progression
		Inferior response
US database	Retrospective analysis of 24,825 patients from the commercial claims database(2000–2018), OPTUM database (2000–2018), and SEER linked database(2007–2016)	*No transplant*
		Older age
		*For Non transplant*
		Older age
		CVS disorders
		Pulmonary disorders
		Renal impairment For transplant
		Liver
European study	Retrospective study of 413 patients treated between 2011 to 2021 from a single Italian centre	Age > 65
		> 2 comorbidities
		No ASCT
		No maintenance
Our study	Retrospective analysis of 244 consecutively diagnosed NDMM patients between January 2017- December 2017 from a single tertiary healthcare centre in Mumbai	Higher Age
		Higher ECOG
		Higher ISS at presentation
		No ASCT

## Conclusion

The attrition rates of NDMM patients are high in an Indian cohort and are significantly associated with poorer survival outcomes.The factors affecting attrition include higher age, poorer ECOG status, and higher ISS at presentation, highlight the association with severe disease at presentation, emphasising the need to initiate most effective therapies upfront to minimize patient drop offs. There is also a need for a prospective registry study to document the attrition rates, potential reasons, and difference in presentation among Indian patients. Additionally, efforts towards raising awareness and promoting earlier diagnosis are essential to improve long term outcomes.
